# Development of In-situ Simulation Lab for Training Gynecology Residents in Basic Laparoscopic and Hysteroscopic Operative Skills

**DOI:** 10.7759/cureus.4385

**Published:** 2019-04-04

**Authors:** Maria M Molina, Thuan H Le, Heather Delaney, Larissa F Weir

**Affiliations:** 1 Obstetrics and Gynecology, Brooke Army Medical Center, Fort Sam Houston, USA; 2 Obstetrics and Gynecology, Malcolm Grow Medical Center, Andrews, USA; 3 Neonatology, Brooke Army Medical Center, San Antonio, USA

**Keywords:** curriculum, simulation, gynecology, medical edcuation, training lab

## Abstract

Introduction: Mounting evidence suggests that practice on simulators leads to improved operative skills and patient safety. With restrictions on resident work hours resulting in less exposure to procedures, simulation is the key to developing operative skills during residency and beyond. Residency programs struggle with implementing a simulation program due to timing and availability of residents. Despite having a large centralized simulation space at our institution, we identified lack of dedicated gynecologic simulation curriculum and simulator accessibility as our greatest barriers to utilizing simulation training in gynecology resident education. We sought to design a space within the resident work area dedicated to gynecologic simulation training with specific curriculum and objectives for each work station based on residency year level.

Methods: We created four workstations in a room within the Ob/Gyn clinic, in close proximity to the resident offices. Two virtual reality simulators, the LapVR (CAE, Montreal, Canada) ($84,996.00) and Simbionix Hystsim (3D Systems, (formerly Simbionix), CO, USA) ($95,741.10), were acquired from our institution’s simulation center and placed in this training space to allow for enhanced resident access. The two other work stations consisted of an FLS trainer box and monitor ($1580) and another low fidelity laparoscopic box trainer and monitor ($450). Specific objectives for each station with corresponding evaluation checklists were written for each residency year level. Dedicated time to meet the written objectives was given to residents each week during their benign gynecology rotation. Supervision and assistance with task completion was provided by staff mentors assigned during those shifts.

Results: Residents who had this simulation lab available to them during their gynecology rotation participated in a minimum of seven hours of simulation time in addition to the time they spent on their own in the lab. These residents felt this was a meaningful increase in the amount of laparoscopic and hysteroscopic simulation exposure by having access to this in-situ GYN Simulation Training Laboratory with a defined gynecologic curriculum. Multiple staff members also took advantage of the simulation lab to practice their skills as well.

Conclusions: We created an in-situ Gyn Simulation Training Lab that allowed for both improved accessibility by the residents and ease of implementation of simulation curriculum into pre-existing resident didactic time. It is our opinion that the time residents spend engaged in surgical simulation will improve surgical skills and confidence thereby enhancing patient safety. Additionally, the creation of this in situ simulation lab assists in meeting the Accreditation Council for Graduate Medical Education (ACGME) requirements for incorporation of simulation into OB/GYN resident education.

## Introduction

Mounting evidence suggests that practice on simulators leads to improved operative skills and patient safety [1]. Dawe et al. has shown that participants who reach proficiency in simulation-based training performed with higher global assessment scores and with fewer errors during patient-based encounters than their counterparts who did not receive simulation training [1]. Following the death of Libby Zion and with the passing of the 405 Bell Regulations in 1989 limiting New York resident to 80-hour work week, the Accreditation Council for Graduate Medical Education (ACGME) officially adopted a similar work hour restriction policy nationwide on July 1, 2003 [2]. As this is a significant change to graduate medical education, residency programs across the country have to be creative in finding approaches to meeting their core competencies while complying with the ACGME rules and regulations. Forty eight percent of these programs have instituted at least one administrative change like implementation of home call or night float system to comply with the duty hour regulations [3]. Jarman et al. noted that with the night float rotation, the projected operations missed by residents were decreased from 202 cases to 107 cases over a span of four years in a general surgery residency [4], however, even with this decrease, a substantial gap still exists. With restrictions on resident work hours resulting in less exposure to procedures, simulation has become critical to developing operative skills during residency and beyond [2].

Residency programs struggle with implementing a simulation program due to timing and availability of residents. Despite having a large centralized simulation space at our institution, we identified lack of dedicated gynecologic simulation curriculum and ease of simulator accessibility as our greatest barriers to utilizing simulation training in gynecology resident education. We sought to design a space within the resident work area dedicated to gynecologic simulation training with specific objectives and curriculum for each work station based on residency year level.

## Materials and methods

**Figure 1:**
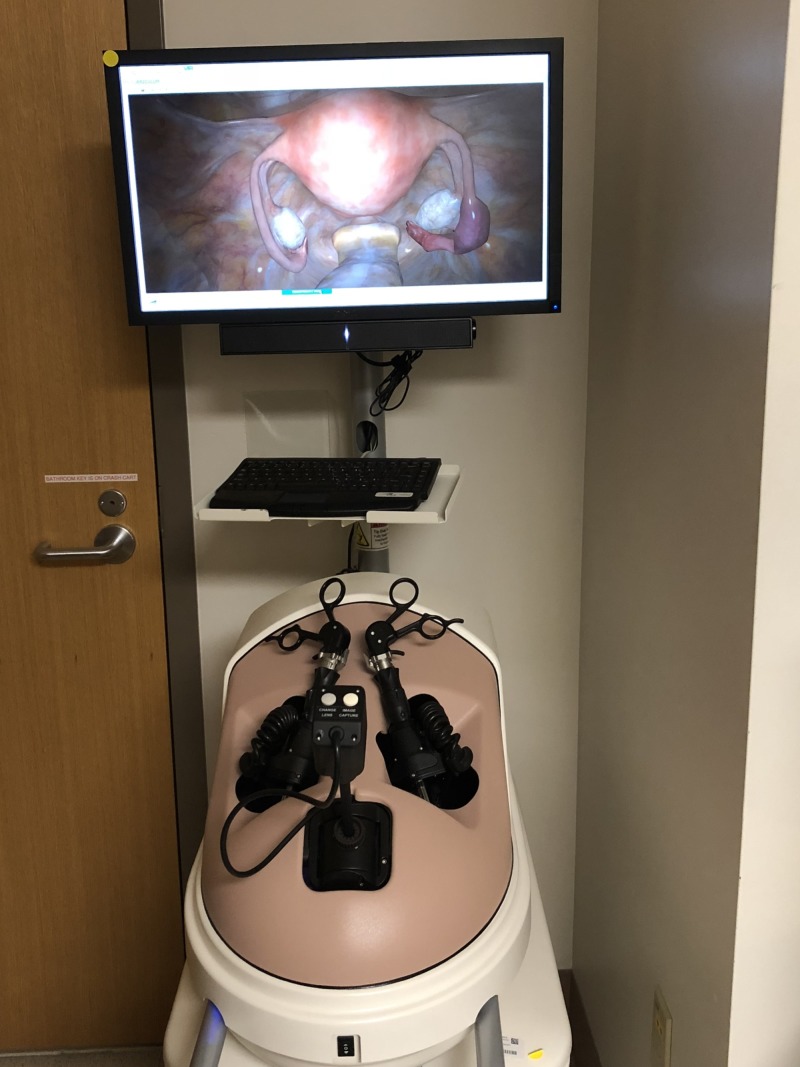
Lap VR (CAE, Montreal, Canada).

**Figure 2:**
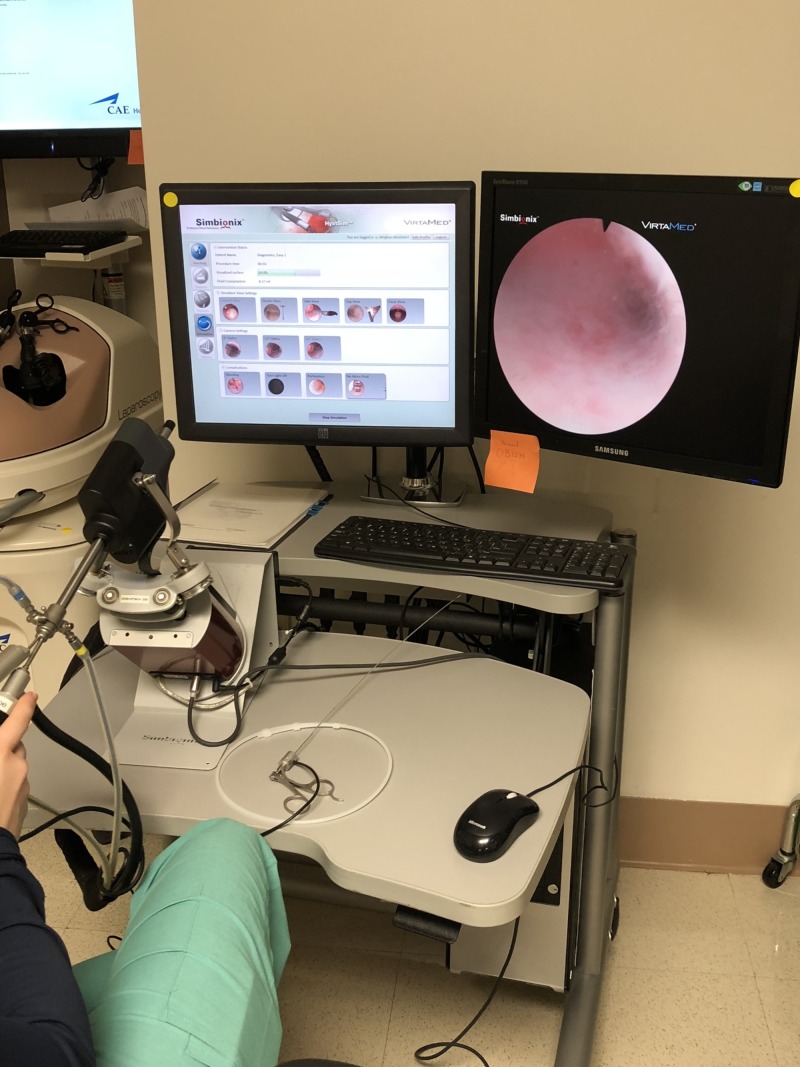
Simbionix Hystsim (3D Systems, (formerly Simbionix), CO, USA).

**Figure 3:**
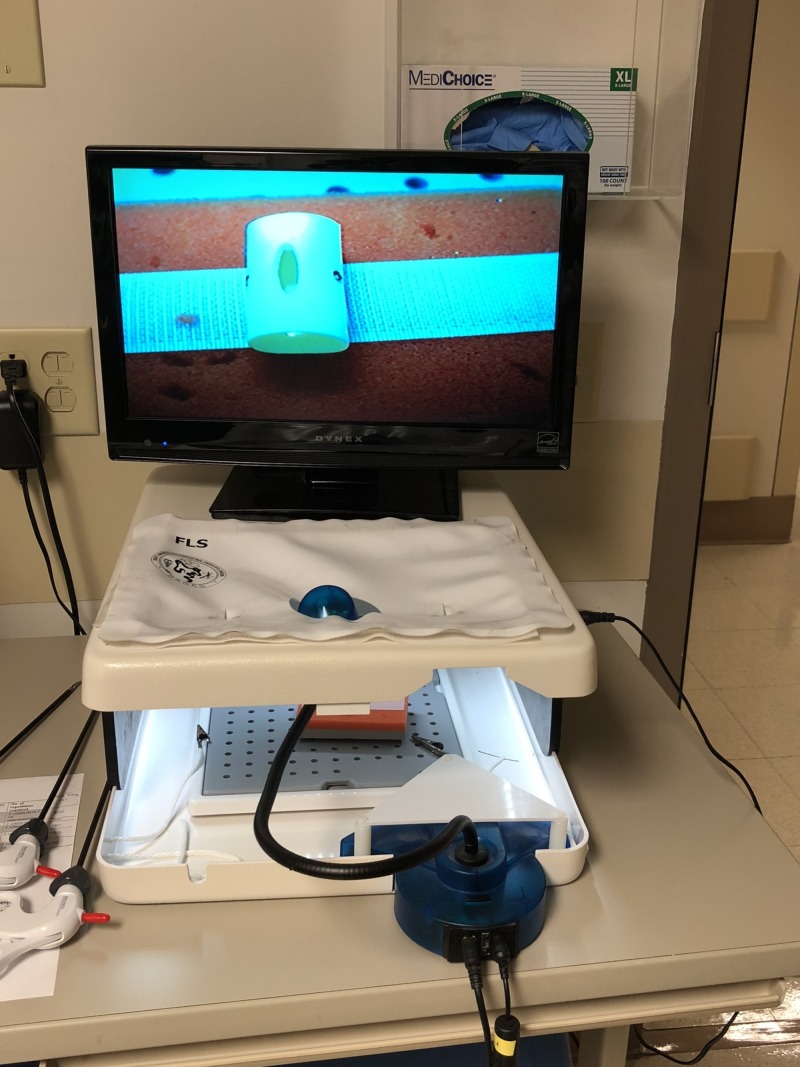
FLS box and monitor.

We created four workstations in a room within the Ob/Gyn clinic, in close proximity to the resident workroom and available 24 hours a day. Two virtual reality simulators, the LapVR (CAE, Montreal, Canada) ($84,996.00) and Simbionix Hystsim (3D Systems, (formerly Simbionix), CO, USA)($95,741.10), were acquired from our institution’s simulation center and placed in this training space to allow for enhanced resident access (Figures 1-2). The other two work stations consisted of FLS trainer boxes and monitors ($1580 each) (Figure 3). Specific objectives for each station with corresponding evaluation checklists were written for each residency year level. Seventy-five minutes of dedicated time to meet the written objectives was given to residents each week during their benign gynecology rotation. Supervision and assistance with task completion was provided by staff mentors assigned during those shifts. Examples of hysteroscopy and laparoscopy goals and objectives as well the laparoscopy box trainer evaluation form for PGY-1 are found in Appendix 1. 

## Results

**Figure 4:**
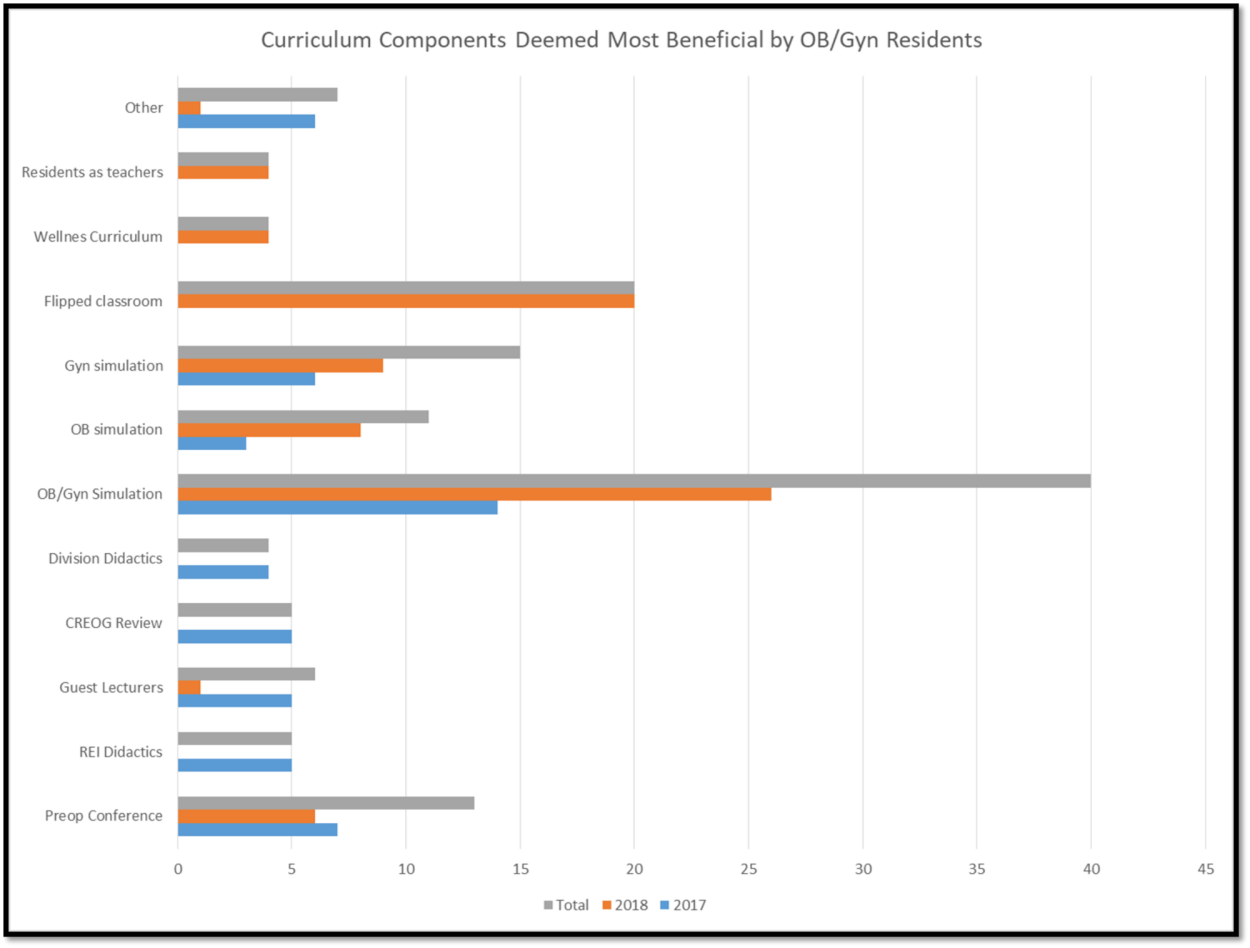
Curriculum components deemed most beneficial by Ob/Gyn residents.

Residents who had this simulation lab available to them during their gynecology rotation had at least seven hours of dedicated simulation time plus any added time they spent on their own in the lab. With this dedicated equipment, curriculum objectives, and the ease of access to this in-situ GYN Simulation Training Laboratory, these residents noted a substantial increase in the amounts of laparoscopic and hysteroscopic simulation time prior to our intervention. They endorsed the realistic experience of both the laporoscopic trainer boxes and the Simbionix Hystsim. Residents reported gaining experience using angled optics, fluid management, and navigation for diagnostic and operative hysteroscopy. With the LapVR, the FLS trainer and the laparoscopic trainer box, they noted an advancement in diagnostic laparoscopy, dexterity skills, fine motor, and spatial relationships in diagnostic and operative laparoscopy. Staff supervision and instant feedback were also critical in honing laparoscopic and hysteroscopic skills. Data from annual resident surveys (aimed at evaluating the entire residency program) were analyzed for 2017 and 2018, following the initiation of our simulation lab and curriculum. When asked to name the top three curriculum components they found most beneficial, 40 out of 41 residents named simulation curriculum as one of their top 3 (Figure 4). One third did not differentiate between obstetric and gynecology simulation, however, one third did specify the curriculum described above as one of their most beneficial. Multiple staff members took advantage of the simulation lab to practice their skills as well.

## Discussion

The standardization of work hours across all residencies with different specialties has resulted in less time spent in both the clinical and surgical setting [5]. Due to time restriction, adjustments have been made and new innovative methods are critical in resident education. A survey conducted by Espey et al. in 2005 indicates that Obstetrics and Gynecology educators perceive a negative impact in resident education and that residents’ surgical volume has diminished secondary to the work hour restrictions [6]. This notion was further validated by Blanchard et al. where they demonstrated a 36.5% decrease in laparoscopy experience and 9.8% hysteroscopy experience after the adoption of the work hour restrictions [7]. Furthermore, laparoscopic surgery is difficult to learn and perform due to the fine motor skills and hand-eye coordination required within the constraints of a two-dimensional image on a video monitor [8]. With duty hour restrictions, the challenges faced in learning and maintaining hysterscopic and laparoscopic proficiency, and the limited training opportunities outside of the operating room, simulation-based training provides a safe and ethical way for trainees to practice surgical skills before entering the OR environment. For the aforementioned reasons, we have created an in-situ Gyn Simulation Training Lab with defined tasks and objectives that improved accessibility by the residents and eased implementation of simulation curriculum into pre-existing resident didactic time. We feel the increase in time residents spend engaged in surgical simulation will improve surgical skills and confidence thereby enhancing patient safety [9]. Additionally, the creation of this in-situ simulation lab assists in meeting the ACGME requirements for incorporation of simulation into OB/GYN resident education.

## Conclusions

Improvements continue to be made to both the curriculum and simulation lab with the recent addition of a laparoscopic tubal ligation, ectopic pregnancy, ovarian cystectomy, and total laparoscopic hysterectomy models. Future studies are needed to objectively measure the improvement in efficiency and safety in the direct care of patients in the operating room since the implementation of the in-situ Gyn Simulation Training Lab.

## Appendices

Hysteroscopy Simulation Goals and Objectives

Goals: Gain experience using angled optics and practice instrument navigation for diagnostic hysteroscopy in a safe environment. Recognition of pathology and various shapes of uterine cavities as well as maintenance of visibility with minimal trauma. 

Objectives:

Complete PGY 1 Course on HystSim trainer with a 75% or more in all sections (1 star or more)

A.     Basic Skills

a.       Access

                       i.      Safely access the uterine cavity using a 30 degree scope

                      ii.      Correctly align the scope for better orientation

                      iii.      Reach the uterine cavity

b.      Distension

                        i.      Safely access the uterine cavity using a 30 degree scope

                       ii.      Correctly identify the in-valve and out-valve

                       iii.      Master the trade-off between max flow and adequate distention

                       iv.      Rinse away fluff and bleeding

                        v.      Clear bubbles by correct positioning and opening of outvalve

c.       Retroverted cavity

                        i.      Safely access a retroverted uterine cavity using a 30 degree scope

                        ii.      Turn the light cord 180 degrees up from the standard entry position to enter retroverted cavity

                       iii.      Reach the cavity

d.      Navigation

                        i.      Safely access the uterine cavity using a 30 degree scope

                        ii.      Learn how to optimally work the 30 degree scope angled for less trauma

                       iii.      Visualize all landmarks by correctly targeting the correct spots

                       iv.      Master the concept of restitution

                        v.      Get a good overview to take a picture

B.     Diagnostic Hysteroscopy

a.       Easy 1-4, Medium 1-4

                         i.      Confirm the correctly placed hysteroscopic device in the uterine cavity by identifying the right and left tubal orifice

                         ii.      Inspect uterine cavity completely by steering the device efficiently over the entire spherical inner surface

                        iii.      Give exact description of visible pathologies

Laparoscopy Simulation Goals and Objectives

Goals:  Practice instrument navigation for diagnostic laparoscopy in a safe environment. Develop technical and dexterity skills need for laparoscopic surgery.   Gain exposure to instruments in a 3-D operative field while looking in a 2-D video thereby improving fine motor and spatial relationships needed for laparoscopy.

Objectives:

Complete PGY 1 Course on LapVR trainer with a pass in all sections.

A. Peg Transfer Module

- Intent is to reach for and grasp a 3D simulated peg with an appropriately modeled instrument and place it into a predetermined hole in the simulated peg board

B. Cutting Module

- Requires appropriate traction with one hand while using the other to cut with scissors

C. Clipping Module

- Requires appropriate traction with one hand while using the other to correctly place two clips to stop blood flow and then cut between the clips

D. Camera Navigation Module

- Requires appropriate maneuvering through all planes of the cavity

Complete the following tasks using laparoscopy box trainers:

A.     Hand-Eye Coordination Task #1

·         Place the open bead container with beads on the peg board in the field of view.

·         Place the top of the container 4-5 spaces away from the bottom

·         Using your dominant had and a dissector, move the 10 beads to the top of the container

·         Repeat the task using your nondominant hand moving the beads back to the container bottom

·         Repeat task this time hand beads from your dominant hand to you nondominant hand then place in container

B.     Hand-Eye Coordination Task #2

·         Place two posts in the peg board base 4-6 spaces apart

·         Place the open bead container with beads on the peg board in the field of view

·         Using your dominant hand and a dissector or grasper, grasp one bead at a time and place over post (total  5)

·         Repeat the task using your nondominant hand

C.     Hand-Eye Coordination Task #3A

·         Make 2 black marks approximately ½ in apart on each side of the rubber band

·         Place posts in peg board 3-4 spaces wider than unstretched length of rubber band

·         Using graspers or dissectors, stretch the rubber band over both posts using one hand to hold the left while stretching the right with the opposite hand

D.     Traction/Counter Traction Task #4

·         Place a wrapped tootsie roll in the trainer

·         Using the graspers or dissectors, grasp opposite ends of the tootsie roll wrapper with the laparoscopic instruments

·         Pull in opposite directions until the wrapper unwinds completely

Laparoscopy Box Trainers Evaluation Form

Instructions for use:  Once you have practiced the tasks multiple times and feel you can “meet expectations” or better for the objectives outlines for a specific task, please have a staff observe and evaluate you using the form below.   Please remind the staff to use a timer.  You only have to complete the tasks assigned to your year level as outlined in the simulation goals and objectives.  Numbers after each task indicate which year level must complete task.

Hand-Eye Coordination Task #1 (1)

1. Total number of dropped pegs  <6

Did not meet expectations (6 or more)        Met expectations (3-5)            Exceeded expectations (0-2)

2. Total number dropped with dominant hand  <3

Did not meet expectations (3 or more)        Met expectations (1-2)            Exceeded expectations (0)

3.  Total number dropped with nondominant hand <3

Did not meet expectations (3 or more)        Met expectations (1-2)            Exceeded expectations (0)

4. Total number dropped during transfer of hands <3

Did not meet expectations (3 or more)        Met expectations (1-2)            Exceeded expectations (0)

5. Time to complete task 6 minutes or less

Did not meet expectations (>6 min)        Met expectations (3-6 min)            Exceeded expectations (<3 min)

Staff Signature­­­­___________________________

Hand-Eye Coordination Task #2 (1,2)

1. Total number of dropped pegs  <4

Did not meet expectations (4 or more)        Met expectations (2-3)            Exceeded expectations (0-1)

2. Total number dropped with dominant hand  <3Did not meet expectations (3 or more)        Met expectations (1-2)            Exceeded expectations (0)

3.  Total number dropped with nondominant hand <3

Did not meet expectations (3 or more)        Met expectations (1-2)            Exceeded expectations (0)

4. Time to complete task 6 minutes or less

Did not meet expectations (>6 min)        Met expectations (3-6 min)            Exceeded expectations (<3 min)

Staff Signature­­­­___________________________

Hand-Eye Coordination Task #3A (1)

1. Total number drops of rubber band <2

Did not meet expectations (2 or more)        Met expectations (1)            Exceeded expectations (0)

2. Time to complete task 1 minute or less

Did not meet expectations (>1 min)       Met expectations (30-60 sec)         Exceeded expectations (<30 sec)

Staff Signature­­­­___________________________

Traction/Counter Traction Task #4 (1)

1. Time to complete task 30 sec or less

Did not meet expectations (>30 sec)        Met expectations (10-30 sec)            Exceeded expectations (<10 sec)

Staff Signature­­­­___________________________

Traction/Counter Traction Task #5 (2)

1. Time to complete task 2 min or less

Did not meet expectations (>2 min)        Met expectations (1-2 min)            Exceeded expectations (<1 min)

Staff Signature­­­­___________________________
